# A latent trajectory analysis of young sexual and gender minorities’ adherence to three rectal microbicide placebo formulations (MTN-035; a randomized crossover trial)

**DOI:** 10.1186/s12889-023-17368-y

**Published:** 2023-12-08

**Authors:** Seul Ki Choi, José Bauermeister, Ryan C. Tingler, Sherri Johnson, Nicole Macagna, Ken Ho, Craig Hoesley, Albert Liu, Noel Kayange, Thesla Palanee-Phillips, Suwat Chariyalertsak, Pedro Gonzales, Jeanna M. Piper, MTN-035 Protocol Team, Abigail Mnemba, Alinafe Kamanga, Annie Munthali, Daniel Gondwe, Linly Seyama, Yamikani Mbilizi, Mary Chadza, Josiah Mayani, Helen Rees, Kerushini Moodley, Krishnaveni Reddy, Andile Twala, Ashleigh Jacques, Tsitsi Nyamuzihwa, Nazneen Cassim, Ana Miranda, Diana Morales, Helen Chapa, Javier Valencia, Milagros Sabaduche, Karina Pareja, Katherine Milagros, Charri Macassi, Pongpun Saokhieo, Veruree Manoyos, Nataporn Kosachunhanan, Piyathida Sroysuwan, Allison Matthews, Amy Player, Andrea Thurman, Carol Mitchell, Christine O’Neill, Christy Pappalardo, Christopher Quan, Cindy Jacobson, Clifford Yip, Craig Hoesley, Danielle Camp, Deon Powell, Devika Singh, Diana Ng, Edward Livant, Elizabeth Brown, Emily Helms, Emily Schaeffer, Faye Heard, Gina Brown, Gustavo Doncel, Holly Gundacker, Hyman Scott, Jackie Fitzpatrick, James Gavel, Jenna Weber, Jennifer Schille, Jessica Webster, Jessica Maitz, Jillian Zemanek, Jim Pickett, Jonathan Lucas, Julie Nowak, Kathleen Dietz, Krissa Welch, Kristine Heath, Lisa Rohan, Lizardo Lacanlale, Lynn Mitterer, Lorna Richards, Marcus Bolton, Mei Song, Naana Cleland, Nicholas Ng, Nnennaya Okey-Igwe, Onkar Singh, Patricia Peters, Rebecca Giguere, Renee Weinman, Roberta Black, Scott Fields, Sharon Riddler, Sharon Hillier, Sherri Karas, Stacey Edick, Sufia Dadabhai, Susan Buchbinder, Taha Taha, Tarana Billups, Teri Senn, Theresa Wagner, Tim McCormick, Yuqing Jiao

**Affiliations:** 1 Department of Family and Community Health, School of Nursing, University of Pennsylvania, 418 Curie Blvd, Room 235L, Philadelphia, PA 19104 USA; 2 FHI 360, Durham, NC USA; 3 Magee-Women’s Research Institute, Pittsburgh, PA USA; 4 Department of Medicine, University of Pittsburgh, Pittsburgh, PA USA; 5 University of Alabama at Birmingham, Birmingham, AL USA; 6 San Francisco Department of Public Health, San Francisco, CA USA; 7 Johns Hopkins University Research Project, Blantyre, Malawi; 8 Faculty of Health Sciences, School of Public Health, Wits Reproductive Health and HIV Institute, University of the Witwatersrand, Johannesburg, South Africa; 9 Department of Epidemiology, School of Public Health, University of Washington, Seattle, USA; 10 Faculty of Public Health, Research Institute for Health Sciences, Chiang Mai University, Chiang Mai, Thailand; 11 IMPACTA, San Miguel, Peru; 12 Division of AIDS/NIAID/NIH, Bethesda, MD USA

**Keywords:** HIV prevention, Pre-exposure prophylaxis, Rectal microbicide, Sexual and gender minorities, Youth, SMS

## Abstract

**Background:**

Rectal microbicides (RM) are biomedical HIV prevention products that aim to prevent or reduce the transmission of HIV and other sexually transmitted infections (STIs). RM modalities may be beneficial for populations who have complex lifestyles, difficulties adhering to pre-exposure prophylaxis (PrEP) regimens, and/or have limited access to care. MTN-035 (DESIRE; Developing and Evaluating Short-Acting Innovations for Rectal Use), a randomized crossover trial, aimed to evaluate the safety and acceptability of, and adherence to, three placebo RM modalities (douche, insert, and suppository) prior to receptive anal intercourse.

**Methods:**

We conducted latent trajectory analysis to identify clusters of individuals who shared similar trajectories in acceptability and adherence for each product (douche, insert, and suppository) over time. We analyzed weekly short messaging service (SMS) use reports for each modality as reported by enrolled sexual and gender minority (SGM) participants.

**Results:**

Two trajectories for each product were identified: a “protocol compliant” trajectory (i.e., at least one product use occasion per week) and “high use” trajectory (i.e., more than three product use occasions per week). Participants with high use were more likely to lack access to PrEP and have higher intentions to utilize RM modalities compared to those who were protocol compliant.

**Conclusions:**

This study highlighted high adherence to RM modalities among SGM. As research into viable HIV prevention modalities continues to evolve, tailored intervention strategies are needed to support the uptake of and adherence to alternative prevention modalities that are behaviorally congruent with targeted users.

**Trial registration:**

NCT03671239 (14/09/2018).

## Background

In the past 30 years, there have been significant strides to prevent and treat human immunodeficiency virus (HIV). In 2021, there were 38.4 million people across the world living with HIV [1]. Of these, 1.5 million were newly diagnosed with HIV in 2021, a 30% decline since 2010 [2]. Innovative biomedical advancements across the HIV prevention continuum (e.g., HIV pre-exposure prophylaxis [PrEP]) have offered new opportunities to curtail HIV incidence, yet new HIV infections have remained high globally due to acceptability, access, uptake, and adherence challenges surrounding these highly effective biomedical prevention tools [3–5].

Rectal microbicides (RM) are biomedical products applied inside the rectum with the goal of preventing or reducing HIV and other sexually transmitted infections (STIs) [6, 7]. RM modalities may be beneficial for populations who have complex lifestyles, difficulties adhering to PrEP regimens, and/or with limited access to care [8, 9]. Findings from previous studies suggest that RM gels might be acceptable among men who have sex with men (MSM) and transgender populations, yet current data suggest lower efficacy levels than daily oral PrEP due to challenges with modality of administration, drug formulation, and adherence [10, 11]. Therefore, researchers and advocates have begun to explore alternative modalities (e.g., douches, suppositories, fast-dissolving inserts) [7, 12].

Alongside the biomedical properties of a drug candidate, the modality through which it is delivered is central to promoting its optimal acceptability and adherence. Within the existing RM literature, there is large variability in responses to product acceptability and adherence. Global behavioral research with MSM and transgender populations has found high hypothetical acceptability of a RM formulated as a douche, insert, or suppository [13–15]. Unfortunately, it is unclear whether these attitudes would persist once MSM and transgender people were able to use them prior to receptive anal intercourse (RAI) with their sexual partners. Therefore, with the goal of supporting the development of behaviorally congruent RM modalities for topical PrEP delivery, the Microbicide Trials Network (MTN) developed MTN-035 (DESIRE; Developing and Evaluating Short-Acting Innovations for Rectal Use) to examine the safety and acceptability of, and adherence to, three modalities (i.e., douches, inserts, and suppositories) among MSM and transgender people living in five different countries across four continents: Malawi, Peru, South Africa, Thailand, and the United States (US).

The goal of this study was to understand how MSM and transgender people enrolled in MTN-035 reported their acceptability of and adherence to each of the three products based on weekly self-reports during each 4-week study product use period. We examined whether participants’ use of each placebo modality (douche, insert, and suppository) changed over time. Given prior research noting the presence of diverse trajectories [16, 17], we hypothesized that we would observe at least two trajectories for each product: one noting an increase in product use over time, and another indicating consistent product use over time. We then examined whether RM product use was associated with participants’ baseline sociodemographic characteristics and sexual behaviors. We hypothesized that participants reporting greater sexual behavior and indicating a greater desire for alternatives to PrEP and condoms would be more likely to be in the increasing RM product use trajectory. While we used placebo modalities in this study and participant were aware of it, different utilization trajectories [18] of placebo modalities may suggest the need to move away from the one-size-fits-all approach in the RM development agenda.

## Methods

### Procedures

#### Sample

Two-hundred and seventeen HIV-uninfected transgender men, transgender women, and cisgender MSM between the ages of 18 and 35 were recruited into the trial. Data collection was conducted between April 2019 and July 2020 in various locations, including Pittsburgh, Pennsylvania; Birmingham, Alabama; and San Francisco, California in the United States, Chiang Mai in Thailand, Lima in Peru, Blantyre in Malawi, and Johannesburg in South Africa.

Participants were recruited from diverse channels, including community-based venues, online platforms, outpatient clinics, universities, and social networking applications. Furthermore, referrals from local research projects and other health and social service providers were also sources for participant recruitment. The study was reviewed and approved by the Institutional Review Boards (IRBs)/Ethics Committees at all participating institutions. This study was submitted and assigned to the number NCT03671239 (14/09/2018) on clincialtrials.gov.

#### Eligibility criteria

The eligibility criteria for this study encompassed the following: 1) men (cis or transgender) and transgender women between 18–35 years old; 2) HIV-1 or HIV-2 uninfected at Screening and Enrollment; 3) in general good health at Screening and Enrollment (e.g., medical and medication history, physical exam including a rectal exam); 4) a reported history of consensual RAI at least three times in the past three months and expectation to maintain at least that frequency of RAI during study participation; 5) For individuals who could get pregnant (transgender men with a female reproductive system), a negative pregnancy test at Screening and Enrollment; 6) For individuals who could get pregnant, use of an effective method of contraception for at least 30 days (inclusive) prior to Enrollment, and intention to use an effective method for the duration of study participation; 7) ability and willingness to provide written informed consent in local language; 8) ability and willingness to provide adequate locator information; 9) availability to return for all study visits and willingness to comply with study participation requirements;10) willingness to not take part in other research studies involving drugs, medical devices, genital or rectal products, or vaccines for the duration of study participation [19]. Participants who met any of the following criteria were excluded from the study: 1) a history of inflammatory bowel disease or anorectal condition that would hinder the placement or assessment of product tolerability; 2) anticipated use of non-study rectally administered products; 3) prior participation in research studies involving rectal products; 4) presence of an active anorectal or reproductive tract infection requiring treatment; 5) symptomatic urinary tract infection (participants with these conditions could be retested during screening and potentially enrolled if resolved); 6) pregnancy or breastfeeding.

#### Screening, enrollment and retention

Prior to enrolling in the study, participants were screened for their eligibility. All participants who were enrolled provided written informed consent. Within the 45-day screening window, participants returned to the clinic for a series of administrative, behavioral, clinical, and laboratory procedures. If clinically necessary, clinical results or treatments for urinary tract infections, genital/reproductive tract infections, sexually transmitted infections, or other findings were provided at each visit. Condoms and lubricant were dispensed to participants during all clinic visits, and they also received HIV/STI risk reduction counseling at screening, enrollment, and during study visits. Consented and enrolled participants were then randomized into one of six sequences, each varying the order in which participants used the study placebo products, with a 1-week wash-out period between each 4-week product use period [19] (Table 1).

**Table 1:** Randomization sequence  order of MTN-035 study

Sequence	N	Period 1 (4 weeks)		Period 2 (4 weeks)		Period 3 (4 weeks)
A	35	Rectal Insert	Washout period (~ 1 week)	Rectal Douche	Washout period (~ 1 week)	Rectal Suppository
B	35	Rectal Douche		Rectal Suppository		Rectal Insert
C	35	Rectal Suppository		Rectal Insert		Rectal Douche
D	35	Rectal Insert		Rectal Suppository		Rectal Douche
E	35	Rectal Douche		Rectal Insert		Rectal Suppository
F	35	Rectal Suppository		Rectal Douche		Rectal Insert

Each participant was monitored for approximately 3.5 months and was required to attend a total of eight visits, which included the Screening and Enrollment visits. A visit was considered to be missed if the participant did not complete any portion of it within the designated visit window. However, if an interim visit was conducted to compensate for the missed regular visit, then the missed regular visit was counted as completed [19].

#### Study procedures

**Fig. 1:**
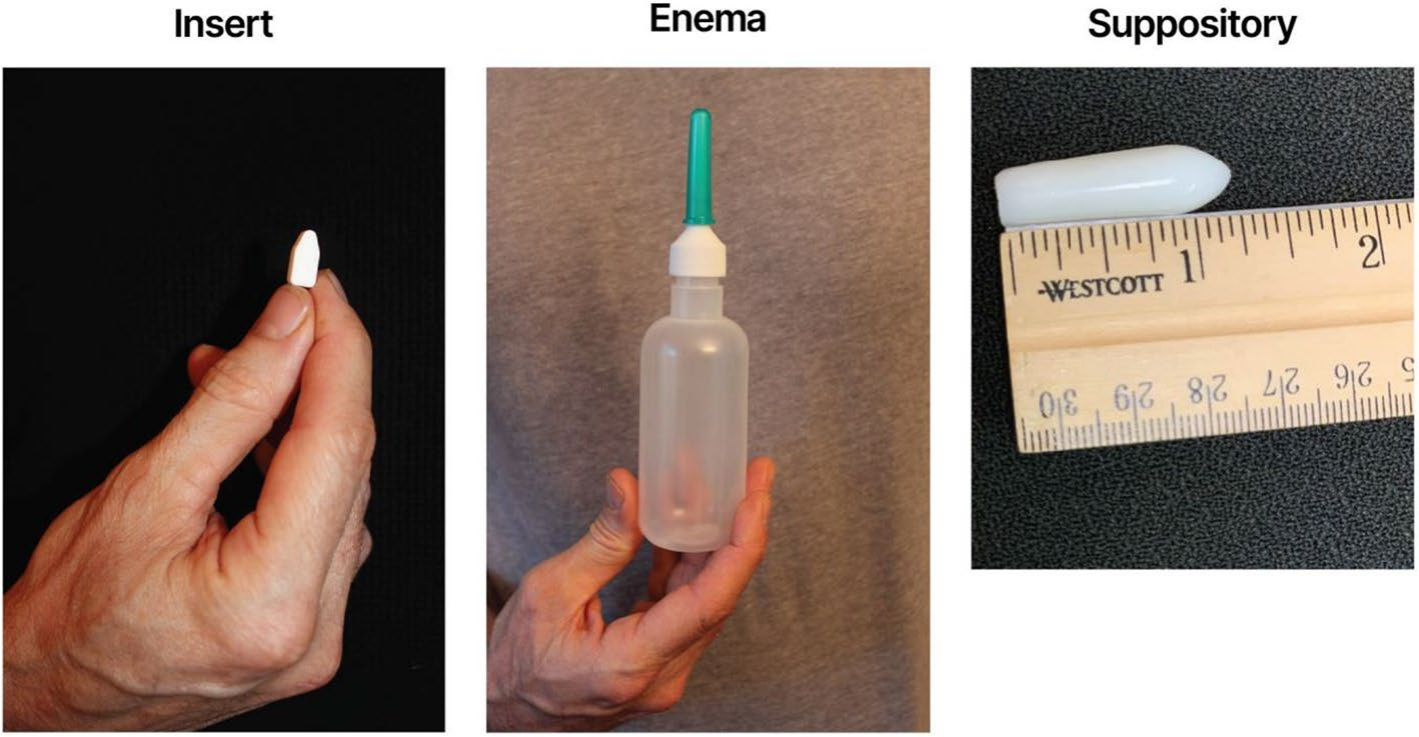
MTN-035 Placebo Study Products

For pericoital rectal administration, each participant was provided with placebo inserts, placebo suppositories, and placebo (water) douche bottles (see Fig. 1). The products were administered in order of the assigned sequence and dispensed prior to each respective product use period. Participants were given instructions to use a single dose of the designated study product within a timeframe of 30 min to 3 h before engaging in RAI, while adhering to their usual pre-RAI routine. They were advised not to exceed more than one dose of the product within a 24-h period. In weeks where participants did not participate in RAI, they were instructed to insert a dose of the product even in the absence of RAI [20]. To ensure accurate administration, participants administered the initial dose of each product themselves under supervision at the clinic.

**Fig. 2:**
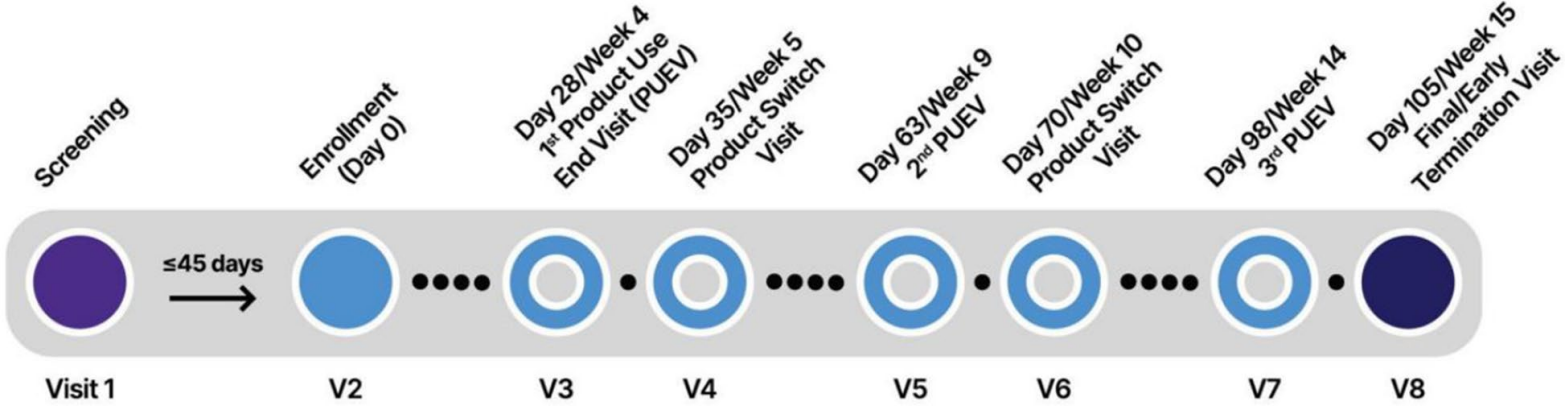
MTN-035 Study Schema

The schedule of participants' study activities can be seen in Fig. 2. During Visit 2, participants received their first rectal product for period 1, based on their assigned sequence. Subsequently, at Visits 3, 5, and 7, participants returned to the clinic for the product use end visits (PUEVs). During these visits, participants underwent various study procedures, including pharyngeal, urine, blood, pelvic (for individuals with a vagina or neovagina), and anorectal tests, if deemed necessary (required at Visit 7). Additionally, participants completed a baseline computer-assisted self-interview (CASI) during their enrollment visit (Visit 2) and at the conclusion of each PUEV (Visits 3, 5, and 7).

After an approximately 7-day wash-out period following study product use periods, participants returned to the clinic to complete Visits 4 and 6 [19]. During these visits, participants completed various study procedures, including pharyngeal, urine, blood, pelvic (for individuals with a vagina or neovagina), and anorectal tests, if necessary. Furthermore, participants self-administered a single dose of the product they were provided and collected the remaining product in their assigned sequence to use for the next four weeks during periods 2 and 3. They were also given instructions on how to use the product [19].

Visit 8 served as the follow-up safety contact and termination visit, during which participants underwent study procedures and received clinical results or treatment for UTIs/RTIs/STIs or other relevant findings [19]. Participant reimbursement followed local guidelines and was approved by the local IRBs/ Ethics Committees prior to the commencement of the study.

To minimize the impact of the COVID-19 pandemic during the data collection period, several measures were implemented, including adjustments to the dispensing of products based on COVID-19 restrictions (i.e., dispensing more than one product at a time) [20]. Among the 78 participants enrolled when the pandemic began, only four did not receive all three products (see Jacobson et al. for details).

### Measures

#### Weekly product use reporting

During the enrollment visit (Visit 2), participants were registered into a short message service (SMS) system. Participants created a personal identification number (PIN) as part of their registration process and selected the time and day of the week when they wanted the SMS system to prompt them to enter their responses. Study staff trained participants on using the system, explained the meaning of abbreviations that would be utilized throughout the study duration, and how to delete their SMS history on their phones to protect their privacy. Participants completed a practice session with study staff before they left the clinic.

During each of their four weeks of product use, participants received weekly messages regarding product use and acceptability on their selected days and times. Participants provided responses based on their most recent report, taking into consideration any potential weeks of missed reporting. Each SMS prompt would begin with a request for the participants’ PIN. Once the PIN was entered correctly, participants were asked to answer four questions: (1) Since your last report, how many times have you used the product? (Range 0–20); (2) Since your last report, how many times did you have RAI? (3) Of those [answer to Q2] times, how many times did you use the product before RAI? (Range 0 to [answer to Q1]; and (4) On a scale from 1–-10, how much did you like the product since your last report (1 = Extremely dislike; 10 = Extremely like). Questions were answered numerically, and responses had to be within feasible ranges. The SMS system was also programmed to accept skip patterns; for example, participants were not prompted to answer Questions 3 or 4 if they reported a 0 for Questions 1 and 2 (i.e., did not use the product and did not have RAI since their last SMS report).

Prior to the PUEVs (Visits 3, 5, and 7), study staff reviewed alongside the participants the SMS data for that study period to ensure accuracy and correct any data that may have been incorrect (e.g., mistyped) or missing due to system (e.g., carrier outages) or user factors (e.g., forgot to complete an entry on a given week). Data that were outside the possible ranges (e.g., exceeding the maximum product dispensed) were corrected to reflect all products used.

The data were then structured on weekly periods. If participants had received more than one message request in a week due to server errors or scheduled visit date changes (i.e., last-minute rescheduling of their PUEV at the clinic or modifications due to COVID-19), we aggregated their entries for that week so that their scores reflected the total sum of the first three questions and averaged their ratings for product acceptability in a given week.

#### Computer Assisted Self-Interview (CASI)

Participants completed a baseline assessment via CASI, which included their sociodemographic characteristics (including their age, level of education, gender identity, sexual identity, and relationship status), sexual behaviors in the past 30 days (e.g., number of sexual partners, number of RAI occasions, and number of condomless RAI occasions). Participants were also asked to report whether they had ever used a douche, insert, or suppository prior to RAI.

The Decisional Balance to Use Condoms Scale [21] was used to examine how participants valued sex with condoms relative to sex without condoms. Participants were asked to answer seven statements. Each statement referred first to “sex with condoms” (e.g., “Sex with condoms makes me feel very connected with my partner”), followed by an identical statement referring to, “sex without condoms” (e.g., “Sex without condoms makes me feel very connected with my partner”). Participants rated each statement using a 4-point scale (1 = Strongly Disagree; 4 = Strongly Agree). Statements were programmed to be randomly presented to each participant to minimize order effects. A net difference score for each pair of the seven statements was calculated, ranging from -3 to 3. Participants’ total decisional balance to use condoms was computed by creating a mean score of these items. Greater positive scores reflect greater benefits/gains associated with sex without condoms. Negative scores reflect greater benefits/gains associated with condom use. Scores hovering close to zero indicate neutrality in the costs and gains associated with sex with or without condoms. The Cronbach’s alpha for the decisional balance scale was 0.92.

We assessed whether participants had ever heard of PrEP and whether they were currently on PrEP, respectively. Participants also noted their future willingness to use each RM modality every time before RAI if it was found to be effective against HIV. Participants answered RM willingness questions for a douche, insert, and suppository modality, respectively, using a 10-point scale (1 = Very Unlikely; 10 = Very Likely).

#### PUEV survey

Acceptability endpoints were based on participants’ responses to the CASI for each product at their respective PUEV (Visits 3, 5 and 7). At the PUEVs, we also asked questions about participants’ most recently used product, including acceptability, using a 10-point scale (1 = Very Unlikely; 10 = Very Likely): how much did you like *RM modality* used as part of this study?

### Data analysis

A total of 217 patients were included in this study. Differences in baseline characteristics between participants who used the SMS system for all three RM products (*n* = 150) and those who did not (*n* = 67) were compared using chi-squared tests for categorical variables and student’s t-tests for continuous variables. Descriptive weekly product use SMS data were calculated.

Then, we developed latent trajectory models to identify clusters of individuals who shared similar trajectories in acceptability and adherence for each product (douche, insert, and suppository) over time. A form of multivariate mixture modeling macro, PROC TRAJ, which employs maximum likelihood to estimate model parameters, was utilized in the study [22]. PROC TRAJ handles missing values and allows participants who did not use the SMS system for all three RM products to be included in the analyses.

For latent trajectory analyses, we included a sample of participants who responded to their SMS system prompts and we tested one- to five-group quadratic trajectory models to find a best-fitting model. We first tested a quadratic trajectory model with one group as a general rule for data with three-time points [23]. If the quadratic parameter of the one-group trajectory model was significant, the analysis for the quadratic trajectory model with two groups was performed. Following these iterative processes, model fit was evaluated based on the Bayesian Information Criterion (BIC), the size of each group, and posterior probabilities (the probability of membership in a specific group). Lower BIC, more than 5% of the sample in each group, and an average probability of ≥ 0.70 for the sample in each trajectory group were used as an indication of a good fit [24]. After finding the best-fitted number of trajectories, we adjusted each trajectory for the best shape (i.e., intercept only, linear, quadratic, or cubic). We compared BIC value to test the improvement of fit. Following the latent trajectory analyses, we created a categorical variable representing a participant’s trajectory group membership for each modality: a protocol compliant trajectory (i.e., at least one product use occasion per week) and high use trajectory (i.e., more than three product use occasions per week).

Finally, we manually created a “no temporal group” (participants who were not included in the latent trajectory analysis due to absence in SMS data) in addition to group membership which were driven by latent trajectory analysis and examined whether different RM group memberships were correlated with participants’ baseline sociodemographic characteristics and sexual behaviors. We also examined associations with acceptability. For participants who responded to acceptability (Q4) in the SMS system, we calculated the average acceptability score over the course of four weeks. We used participants’ acceptability scores from their PUEV survey if participants had not responded to the acceptability prompt in the SMS system. Fishers’ exact tests were used for categorical correlates and student’s t-tests were used for continuous correlates. In addition, multinomial logistic regression models were conducted to estimate the association between each trajectory and correlates. All analyses were conducted using SAS 9.4 (SAS Institute Inc., 2013).

## Results

### Sample description

Table 2 summarizes descriptive information about the participants in detail. The mean age of the sample was 24.9 (SD 4.6) years. Less than half of the participants lived in the US (44.2%) and had an education level of college degree or above (44.2%). More than half of the sample identified as male (67.4%) and homosexual (71.9%). Some participants had used RM products in the past. Participants noted that douches were the most frequently used product (65.4%), followed by suppositories (19.7%), and inserts (5.9%).

**Table 2:** Comparison of MTN-035 participants’ baseline characteristics between those who provided SMS data on all three modalities and those did not provide SMS data for all three modalities

	Total (*n* = 217)	SMS data on all three modalities (*n* = 150)	SMS data missing for one or more modalities (*n* = 67)	
	N (%)/ Mean (SD)	N (%)/ Mean (SD)	N (%)/ Mean (SD)	*p*-value*
*Demographic characteristics*				
Site, n (%)				** < .0001**
Pittsburgh, US	33 (15.2%)	29 (19.3%)	4 (6.0%)	
Birmingham, US	33 (15.2%)	28 (18.7%)	5 (7.5%)	
San Francisco, US	30 (13.8%)	30 (20.0%)	0 (0%)	
Lima, Peru	30 (13.8%)	14 (9.3%)	16 (23.9%)	
Chiang Mai, Thailand	30 (13.8%)	19 (12.7%)	11 (16.4%)	
Blantyre, Malawi	31 (14.3%)	21 (14.0%)	10 (14.9%)	
Johannesburg, SA	30 (13.8%)	9 (6.0%)	21 (31.3%)	
Age, mean (SD)	24.9 (4.6)	25.4 (4.8)	23.7 (4.0)	**.012**
Education, n (%)				**.027**
Less than college	121 (55.8%)	76 (50.7%)	45 (67.2%)	
More than college	96 (44.2%)	74 (49.3%)	22 (32.8%)	
Sex Assigned at Birth, n (%)				.669
Male	211 (97.2%)	145 (97.7%)	66 (98.5%)	
Female	6 (2.8%)	5 (3.3%)	1 (1.5%)	
Gender identity, n (%)				.180
Male	146 (67.6%)	107 (71.8%)	39 (58.2%)	
Female	8 (3.7%)	5 (3.4%)	3 (4.5%)	
Transgender Male	8 (3.7%)	4 (2.7%)	4 (6.0%)	
Transgender Female	30 (13.9%)	20 (13.4%)	10 (14.9%)	
Gender Queer	14 (6.5%)	6 (4.0%)	8 (11.9%)	
Other Gender	10 (4.6%)	7 (4.7%)	3 (4.5%)	
Sexual identity, n (%)				**.004**
Gay, Lesbian, Homosexual	156 (71.9%)	112 (74.7%)	44 (65.7%)	
Straight	12 (5.5%)	8 (5.3%)	4 (6.0%)	
Bisexual	34 (15.7%)	26 (17.3%)	8 (11.9%)	
Other	15 (6.9%)	4 (2.7%)	11 (16.4%)	
Relationship status, n (%)				.142
In a relationship	94 (43.3%)	70 (46.7%)	24 (35.8%)	
Single	123 (56.7%)	80 (53.3%)	43 (64.2%)	
*Behavior Measures*				
Number of sexual partners, mean (SD)	3.0 (2.6)	3.0 (2.6)	3.0 (2.3)	.874
Number of anal sex occasions, mean (SD)	7.9 (15.8)	8.5 (18.1)	6.6 (8.1)	.455
Condomless anal sex, n (%)				**.035**
No	136 (62.7%)	87 (58.0%)	49 (73.1%)	
Yes	81 (37.3%)	63 (42.0%)	18 (26.9%)	
Ever douche, n (%)				.164
No	75 (34.6%)	47 (31.3%)	28 (41.8%)	
Yes	142 (65.4%)	103 (68.7%)	39 (58.2%)	
Ever insert, n (%)				.311
No	175 (94.1%)	124 (95.4%)	51 (91.1%)	
Yes	11 (5.9%)	6 (4.6%)	5 (8.9%)	
Ever suppository, n (%)				.817
No	114 (80.3%)	82 (79.6%)	32 (82.1%)	
Yes	28 (19.7%)	21 (20.4%)	7 (18.0%)	
Decisional Balance for Condom use, mean (SD)	0.2 (1.3)	0.04 (1.2)	0.6 (1.3)	**.004**
Ever heard about PrEP, n (%)				.303
No	31 (14.3%)	19 (12.7%)	12 (17.9%)	
Yes	186 (85.7%)	131 (87.3%)	55 (82.1%)	
Currently on PrEP, n (%)				**.010**
No	151 (69.6%)	96 (64.0%)	55 (82.1%)	
Yes	66 (30.4%)	54 (36.0%)	12 (17.9%)	
Willingness to use each RM modality every time before RAI, mean (SD)				
Douche	7.3 (2.6)	7.0 (2.7)	7.9 (2.4)	**.024**
Insert	7.0 (2.6)	6.7 (2.6)	7.7 (2.5)	**.007**
Suppository	7.0 (2.7)	6.8 (2.7)	7.5 (2.5)	.062

*comparing difference between participants who reported SMS data on all three RM modalities and participants who did not report SMS data on all three modalities

Sixty-seven participants did not use the SMS system for at least one RM product due to SMS restrictions placed by participants’ mobile carriers (*n* = 23), or due to low compliance from participants (e.g., *n* = 22 did not complete SMS for one product sequence; *n* = 22 did not complete for two product sequences. Participants in Johannesburg, where SMS cost (two rands [about 10 cents USD] per SMS), were less likely to report SMS data for all three RM modalities compared to participants in the US, where SMS are free (*p* < 0.0001). Also, participants who reported all three RM SMS data were more likely to be older, have a college degree, self-identify as gay, engage in condomless anal intercourse, have lower decisional balance for condom use, and have a lower willingness to use douche and insert modalities every time before RAI (all *p* < 0.05).

### Weekly product use report

We summarized four questions that were asked through the SMS system tracking weekly product use (see Table 3). The number of product use occasions, the number of RAI occasions, and the number of product use during RAI occasions increased with time, regardless of the types of the RM product. While acceptability was similar across different products and across different time points, it is possible that usage increased as participants became more familiar with the products. Future research is needed to understand the relationship between RM acceptability and changes in utilization over time.

**Table 3:** Summary of SMS data for all three modalities over 4 weeks

	Douche				Insert				Suppository			
	Week 1 (*n* = 124)	Week 2 (*n* = 131)	Week 3 (*n* = 124)	Week 4 (*n* = 86)	Week 1 (*n* = 124)	Week 2 (*n* = 124)	Week 3 (*n* = 128)	Week 4 (*n* = 79)	Week 1 (*n* = 127)	Week 2 (*n* = 130)	Week 3 (*n* = 119)	Week 4 (*n* = 81)
Q1, mean (SD)	1.69 (1.35)	2.52 (3.14)	2.98 (3.38)	3.23 (3.95)	1.75 (1.46)	2.74 (2.75)	2.95 (3.87)	3.76 (4.67)	1.98 (1.72)	2.68 (2.69)	2.76 (3.17)	2.41 (2.73)
Q2, mean (SD)	1.46 (1.75)	2.34 (3.25)	3.08 (6.25)	2.57 (3.66)	1.23 (1.44)	2.19 (2.39)	2.50 (4.06)	2.82 (3.94)	1.63 (2.01)	2.35 (2.82)	2.62 (3.22)	2.21 (2.86)
Q3, mean (SD)	1.12 (1.36)	1.96 (3.16)	2.16 (2.96)	2.01 (3.04)	0.89 (1.12)	1.77 (2.02)	2.06 (3.49)	2.36 (3.67)	1.25 (1.51)	1.82 (2.36)	2.20 (3.14)	1.77 (2.71)
Q4, mean (SD)	6.40 (3.04)	6.80 (2.64)	6.77 (2.68)	6.56 (2.83)	6.24 (2.72)	6.50 (2.58)	6.46 (2.84)	6.13 (3.03)	6.10 (2.88)	6.57 (2.50)	6.29 (2.84)	6.08 (3.10)

Q1 = the number of product usages since last report (range 0–20); Q2 = the number of RAI occasions since last report; Q3 = the number of product usages before RAI (range 0-Q1); Q4 = product acceptability since last report (range 0–10)

### Trajectories of RM product utilization

**Fig. 3:**
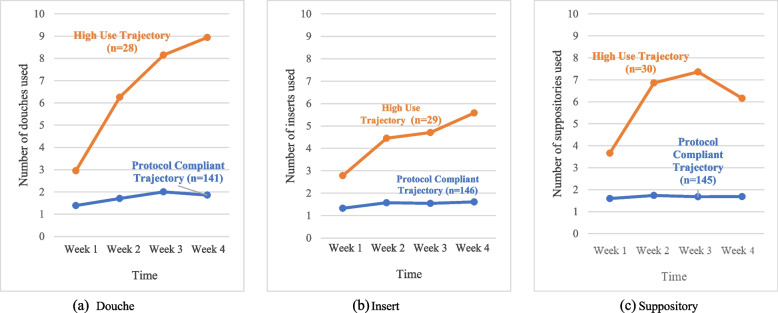
Participants’ average number of products use over time across the **a** douche, **b** insert, and **c** suppository modalities

We iteratively compared models with the increasing number of groups and omitting/adding parameters. Two-group trajectory solutions were selected for all three RM products (douche, insert, suppository); lowest BIC (BIC = -915.63; -895.53; -887.72, respectively), at least 18% of the sample in the smallest group. Non-significant quadratic and linear terms were omitted from the first and second trajectory. The results of the two-group solutions are shown in Tables 4 and 5 and Fig. 3.

**Table 4:** Estimates of two-group trajectory models for three RM modalities use

Group	Parameter	Douche			Insert			Suppository		
		Estimate (SE)	t	*P*-value	Estimate (SE)	t	*P*-value	Estimate (SE)	t	*P*-value
Class 1 Protocol Compliant	Intercept	0.62 (0.06)	9.51	< .0001	0.60 (0.06)	9.87	< .0001	0.51 (0.05)	10.25	< .0001
	Linear	1.09 (0.44)	2.49	.013	1.03 (0.40)	2.58	.010	ns		
	Quadratic	ns			ns			ns		
Class 2 High Use	Intercept	2.27 (0.10)	23.47	< .0001	2.30 (0.08)	28.75	< .0001	1.89 (0.07)	25.71	< .0001
	Linear	1.70 (0.60)	2.83	.005	2.61 (0.60)	4.38	< .0001	1.64 (0.48)	3.43	.001
	Quadratic	-20.15 (4.92)	-4.10	< .0001	-14.03 (4.63)	-3.03	.003	ns		

ns = omitted in the modeling due to non-significance

**Table 5:** Model fit statistics of two-group trajectory models for three RM modalities use

	Douche	Insert	Suppository
BIC	-915.63	-895.53	-887.72
AIC	-903.41	-883.26	-878.55

*BIC* Bayesian Information Criterion, *AIC* Akaike Information Criterion

#### Douche

The ‘protocol compliant trajectory’ accounted for most of the sample (*n* = 141) and was characterized by having an average of one product use per week (see Fig. 3a). The ‘high use trajectory’ (*n* = 28) was characterized by greater douche utilization over time (range 2.95–8.83).

Douche trajectory group membership was significantly associated with living in the US, education level, age, decisional balance for condom use, and current PrEP status (see Table 6). The ‘high use trajectory’ and ‘no temporal data’ groups had more participants who did not live in the US compared to the ‘protocol compliant trajectory’ (75.0%, 91.8%, and 39.3%, respectively; *p* < 0.001). Also, participants in the ‘protocol compliant’ trajectory were older compared to those in the ‘no temporal data’ group (Mean 25.5 SD 4.7 vs. Mean 23.5 SD 3.9; *p* = 0.026). The ‘protocol compliant trajectory’ group was more likely to have more than a college education (52.9%; *p* = 0.003) and be on PrEP (40%; *p* < 0.001) compared to the other two groups. In addition, decisional balance for condom use (Mean -0.04 SD 1.2; *p* = 0.001) and intentions to douche with every RAI occasion (Mean 6.9 SD 2.7; *p* = 0.006) were lower among participants in the ‘protocol compliant trajectory’ group. Finally, the acceptability of the product was higher among the ‘high use trajectory’ group, followed by ‘no temporal data’ group and ‘protocol compliant trajectory’ group (*p* = 0.001).

**Table 6:** Associations between participants’ trajectories of douche use and their baseline characteristics

	Douche Trajectory			
	Class 1 – Protocol Compliant (*n* = 141)	Class 2 – High Use (*n* = 28)	No temporal data^a^ (*n* = 48)	*P*-value †
*Number of douches used, n (%)*				
Time 1	1.41 (0.9)	2.95 (2.2)	-	
Time 2	1.69 (1.0)	6.2 (5.8)	-	
Time 3	2.01 (1.4)	8.0 (5.7)	-	
Time 4	1.75 (1.2)	8.83 (5.5)	-	
*Demographic characteristics*				
US, n (%)				** < .001**
No	55 (39.3)	21 (75.0)	45 (91.8)	
Yes	85 (60.7)	7 (25.0)	4 (8.2)	
Age, mean (SD)	25.5 (4.7)	24.2 (4.9)	23.5 (3.9)	**.026**
Education, n (%)				**.003**
Less than college	66 (47.1)	21 (75.0)	34 (69.4)	
More than college	74 (52.9)	7 (25.0)	15 (30.6)	
Gender identity, n (%)				.115
Other	40 (29.0)	8 (28.6)	22 (44.9)	
Male	98 (71.0)	20 (71.4)	27 (55.1)	
Sexual identity, n (%)				.090
Other	33 (23.6)	12 (42.9)	16 (32.7)	
Homosexual	107 (76.4)	16 (57.1)	33 (67.3)	
Relationship status, n (%)				.552
In a relationship	64 (45.7)	12 (42.9)	18 (36.7)	
Single	76 (54.3)	16 (57.1)	31 (63.3)	
*Behavior Measures*				
Number of sexual partners, mean (SD)	3.06 (2.6)	2.64 (2.6)	3.02 (2.7)	.738
Condomless anal intercourse, n (%)				.181
No	82 (58.6)	18 (64.3)	36 (73.5)	
Yes	58 (41.4)	10 (35.7)	13 (26.5)	
Ever used a douche, n (%)				.845
No	47 (33.6)	11 (39.3)	17 (34.7)	
Yes	93 (66.4)	17 (60.7)	32 (65.3)	
Decisional Balance for Condom use, mean (SD)	-0.04 (1.2)	0.85 (1.4)	0.53 (1.2)	**.001**
Ever heard of PrEP, n (%)				.840
No	19 (13.6)	5 (17.9)	7 (14.3)	
Yes	121 (86.4)	23 (82.1)	42 (85.7)	
Currently on PrEP, n (%)				** < .001**
No	84 (60.0)	26 (92.9)	41 (83.7)	
Yes	56 (40.0)	2 (7.1)	8 (16.3)	
Willingness to use a RM douche every time before RAI, mean (SD)	6.9 (2.7)	8.1 (2.4)	8.1 (2.2)	**.006**
*Acceptability*				
Douche (*n* = 169)^b^, mean (SD)	6.3 (2. 6)	8.3 (1.5)	7.8 (3.1)	**.001**

^a^We created No Temporal Data group for participants who do not have SMS data. †Comparing differences between Class1, Class 2, and No temporal data. ^b^For participants who responded about acceptability in SMS system, we calculated average acceptability score over four weeks. For participants who did not respond about acceptability in SMS system, we pulled acceptability from PUEV survey

#### Insert

Most of the sample (*n* = 146) was grouped on the ‘protocol compliant trajectory’ (see Fig. 3b). There were no significant changes across four-time points (range 1.45–-2.02). The second group (*n* = 29), characterized as the ‘high use trajectory,’ reported increased insert utilization over time (range 3.24–11.86).

Living in the US, educational level, age, gender, sexual identity, and current PrEP status were associated with insert trajectory group memberships (see Table 7). The ‘protocol compliant trajectory’ group had more participants who lived in the US followed by the ‘high use trajectory’ and ‘no temporal data’ groups (57.2%, 37.9%, and 4.6%, respectively; *p* < 0.001). Participants in the ‘protocol compliant trajectory’ group had lower educational attainment compared to other groups (83.8%; *p* = 0.008). The ‘protocol compliant trajectory’ group had more participants who self-identified as male than the other two groups (72.7%; *p* = 0.033), had more participants who identified as homosexual than those in the ‘high use trajectory’ group (76.6% vs. 58.6%; *p* = 0.0495). PrEP uptake was significantly higher in the ‘protocol compliant trajectory’ group compared to the other two groups (38.6%; *p* = 0.002). There were no significant differences in acceptability across groups.

**Table 7:** Associations between participants’ trajectories of insert use and their baseline characteristics

	Insert Trajectory			
	Class 1 – Protocol Compliant (*n* = 146)	Class 2 – High Use (*n* = 29)	No temporal data^a^ (*n* = 43)	*P*-value †
*Number of inserts used, n (%)*				
Time 1	1.45 (1.1)	3.24 (2.1)	-	
Time 2	1.69 (1.1)	6.92 (3.4)	-	
Time 3	1.79 (1.2)	9.2 (6.5)	-	
Time 4	2.02 (1.7)	11.86 (5.6)	-	
*Demographic characteristics*				
US, n (%)				** < .001**
No	62 (42.8)	18 (62.1)	41 (95.4)	
Yes	83 (57.2)	11 (37.9)	2 (4.6)	
Age, mean (SD)	25.44 (4.7)	23.48 (4.5)	23.88 (4.1)	**.039**
Education, n (%)				**.008**
Less than college	72 (49.7)	24 (82.8)	25 (58.1)	
More than college	73 (50.3)	5 (17.2)	18 (41.9)	
Gender, n (%)				**.033**
Other	39 (27.3)	10 (34.5)	21 (48.8)	
Male	104 (72.7)	19 (65.5)	22 (51.2)	
Sexual identity, n (%)				.084
Other	34 (23.5)	12 (41.4)	15 (34.9)	
Homosexual	111 (76.6)	17 (58.6)	28 (65.1)	
Relationship status, n (%)				.763
In a relationship	63 (43.5)	14 (48.3)	17 (39.5)	
Single	82 (56.6)	15 (51.7)	26 (60.5)	
*Behavior Measures*				
Number of sexual partners, mean (SD)	3.03 (2.5)	3.29 (3.00)	2.66 (2.5)	.601
Condomless anal intercourse, n (%)				.083
No	90 (62.1%)	14 (48.3%)	32 (74.4%)	
Yes	55 (37.9%)	15 (51.7%)	11 (25.6%)	
Ever used an insert, n (%)				.298
No	119 (95.2)	26 (96.3)	30 (88.2)	
Yes	6 (4.8)	1 (3.7)	4 (11.8)	
Decisional Balance for Condom use, mean (SD)	0.11 (1.2)	0.36 (1.6)	0.42 (1.3)	.334
Ever heard of PrEP, n (%)				.290
No	17 (11.7)	5 (17.2)	9 (20.9)	
Yes	128 (88.3)	24 (82.8)	34 (79.1)	
Currently on PrEP, n (%)				**.002**
No	89 (61.4)	25 (86.2)	37 (86.1)	
Yes	56 (38.6)	4 (13.8)	6 (13.9)	
Willingness to use a RM insert every time before RAI, mean (SD)	6.7 (2.6)	7.3 (2.7)	7.8 (2.6)	.067
*Acceptability*				
Insert (*n* = 168)^b^, mean (SD)	6.1 (2.4)	7.1 (2.5)	6.7 (3.6)	.153

^a^We created temporal group for participants who do not have SMS data. †Comparing differences between Class1, Class 2, and No temporal data. ^b^For participants who responded about acceptability in SMS system, we calculated average acceptability score over four weeks. For participants who did not respond about acceptability in SMS system, we pulled acceptability from PUEV survey

#### Suppository

The first group consisted of those in the ‘protocol compliant trajectory’ (*n* = 145), with similar utilization across the four weeks (see Fig. 3c). The second group (*n* = 30), the ‘high use trajectory’ group, reported an increasing trend in utilization from time 1 (3.66) to time 3 (7.36), then a small decline of utilization from time 3 to time 4 (6.16).

The ‘protocol compliant trajectory’ group had more participants living in the US compared to the other two groups (59%; *p* < 0.001; see Table 8). The ‘high use trajectory’ group had more participants with lower educational attainment compared to those in the ‘protocol compliant trajectory’ group (76.7% vs. 50%; *p* = 0.027). The ‘protocol compliant trajectory’ group had a greater number of participants who had heard of PrEP (91%; *p* = 0.010) compared to the other two groups. Also, participants’ decisional balance for condom use (mean 0.00; SD 1.2; *p* = 0.005) was lower among participants in the ‘protocol compliant trajectory’ group. Participants in the ‘protocol compliant trajectory’ group had a larger number of participants who were on PrEP, followed by those with no temporal data, and then participants in the 'high use trajectory' group (39.6%, 18.6%, and 3.3%, respectively; *p* = 0.001). Participants’ intention to use a RM suppository every time before RAI was higher among the ‘high use trajectory’ group compared to the ‘protocol compliant trajectory’ group (*p* = 0.010). Overall suppository acceptability was lower in the ‘protocol compliant trajectory’ group compared to the other two groups (*p* = 0.003).

**Table 8:** Associations between participants’ trajectories of suppository use and their baseline characteristics

	Suppository Trajectory			
	Class 1 – Protocol compliant (*n* = 145)	Class 2 – High use (*n* = 30)	No temporal data^a^ (*n* = 43)	*P*-value †
*Number of suppositories used, n (%)*				
Time 1	1.60 (1.17)	3.77 (2.62)	-	
Time 2	1.72 (1.11)	6.96 (3.39)	-	
Time 3	1.68 (1.26)	7.55 (4.46)	-	
Time 4	1.68 (1.44)	6.23 (4.35)	-	
*Demographic characteristics*				
US, n (%)				** < .001**
No	59 (41.0)	25 (83.3.)	37 (86.1)	
Yes	85 (59.0)	5 (16.7)	6 (13.9)	
Age, mean (SD)	25.16 (4.6)	24.79 (5.2)	24.00 (4.2)	.357
Education, n (%)				**.027**
Less than college	72 (50.0)	23 (76.7)	26 (60.5)	
More than college	72 (50.0)	7 (23.3)	17 (39.5)	
Gender, n (%)				.120
Other	40 (28.0)	13 (44.8)	17 (39.5)	
Male	103 (72.0)	16 (55.2)	26 (60.5)	
Sexual identity, n (%)				.243
Other	36 (25.0)	12 (40.0)	13 (30.2)	
Homosexual	108 (75.0)	18 (60.0)	30 (69.8)	
Relationship status, n (%)				.672
In a relationship	62 (43.1)	15 (50.0)	17 (39.5)	
Single	82 (56.9)	15 (50.0)	26 (60.5)	
*Behavior Measures*				
Number of sexual partners, mean (SD)	3.16 (2.7)	2.64 (2.4)	2.68 (2.3)	.436
Condomless anal intercourse, n (%)				.437
No	86 (59.7%)	20 (66.7%)	30 (69.8%)	
Yes	58 (40.3%)	10 (33.3%)	13 (30.2%)	
Ever used a suppository, n (%)				.119
No	75 (78.1)	11 (68.8)	28 (93.3)	
Yes	21 (21.9)	5 (31.3)	2 (6.7)	
Decisional Balance for Condom use, mean (SD)	0.00 (1.2)	0.72 (1.3)	0.54 (1.4)	.005
Ever heard of PrEP, n (%)				.010
No	13 (9.0)	8 (26.7)	10 (23.3)	
Yes	131 (91.0)	22 (73.3)	33 (76.7)	
Currently on PrEP, n (%)				**.001**
No	87 (60.4)	29 (96.7)	35 (81.4)	
Yes	57 (39.6)	1 (3.3)	8 (18.6)	
Willingness to use RM suppository every time before RAI, mean (SD)	6.6 (2.7)	8.2 (2.2)	7.3 (2.5)	**.010**
*Acceptability*				
Suppository (*n* = 165)^b^, mean (SD)	6.0 (2.3)	7.6 (2.4)	7.5 (2.2)	**.003**

^a^We created temporal group for participants who do not have SMS data. †Comparing differences between Class1, Class 2, and No temporal data. ^b^For participants who responded about acceptability in SMS system, we calculated average acceptability score over four weeks. For participants who did not respond about acceptability in SMS system, we pulled acceptability from PUEV survey

## Discussion

The findings from this study underscore the high acceptability of and adherence to RM products for HIV prevention among participants living across six different countries. Participants’ adherence complied with the protocol expectations (i.e., participants were to use the product at least once a week if they did not engage in RAI). Using latent class trajectory analyses, we were able to understand differences in participants’ RM use trajectories using their SMS self-reports. Similar to prior research on rectal gel use for HIV prevention among transgender women and MSM [17], we found our sample could be characterized based on their product use over time. We observed two group-based trajectories across the three modalities, with the two trajectories being distinguished by the quantity of product usage reported per week. In our study, participants were instructed to insert one dose of the product prior to RAI or one dose without sex if participants did not engage in RAI in a given week. Reflecting the study design, the ‘protocol compliant trajectory’ group used the product at least once a week. Participants in the ‘high use trajectory’ group tended to increase their average product use over time. These findings underscore the potential for these three modalities as RM vehicles, offering alternatives to RM gel candidates and existing systemic PrEP products.

The differences observed across trajectories suggest moving away from one-size-fits-all approaches, as participants’ frequency of use over time varied based on their sociodemographic and behavioral characteristics. For example, participants in the ‘protocol compliant trajectory’ group were more likely to live in the US, where PrEP is more readily available, and to self-report PrEP use than those in the ‘high use trajectory’ group. Participants in the ‘high use trajectory’ group, on the other hand, reported higher intentions to use RM in the future if it protected against HIV and were characterized as having greater educational attainment than peers in the ‘protocol compliant trajectory’ group. Given these findings, there is a need to address the variability in product use over time as future RM and other HIV prevention trials are undertaken. Future research should invest in the development and testing of interventions that account for individuals’ variability in their sociodemographic characteristics (e.g., education, geographic region where they live, access to PrEP), and leverage participants’ psychosocial constructs (e.g., intentions to use RM in the future) to promote product use and adherence.

This study had several strengths and limitations deserving mention. First, this is the first study to examine the systematic use of these three promising placebo RM modalities for rectal drug delivery prior to RAI and examine participants’ experiences with all three products. Examining each product’s use in real life contexts strengthens the social validity of our findings and the potential use for these three modes of delivery in the future. As a limitation, however, we were unable to collect SMS data from all participants. While we included these participants as their own group in our analyses, it is unclear what trajectory they would have belonged to had they provided SMS data. Second, due to the use of placebo RM modalities, it is unclear whether the trajectory and acceptability of placebo RM modalities match those of actual RM modalities had an active drug been administered. Third, our ability to recruit and retain a large sample of young SGM living in geographically and socio-politically diverse countries is noteworthy and strengthens the generalizability of our findings to diverse contexts. Unfortunately, we are unable to run country-specific analyses given the limited sample size within each country. Given the variation in structural and cultural contexts, such as the state of their healthcare systems, social norms, perceptions of HIV and various prevention methods, and acceptance of SGM, future research examining how these contextual factors influence participants’ use and adherence to study products is warranted. Fourth, the prospective collection of weekly SMS-facilitated data from participants allowed for the modeling of the product use trajectories. While self-reported data may be biased because of recall and social desirability issues, we believe that recall and social desirability biases were minimized through the on-site training using the SMS system, including how to protect participants’ privacy, and participants’ ability to verify their entries at each PUEV. Moreover, this analysis examines product use with RAI and at least once per week if participants did not have RAI in a given week. At present, we are unable to assess whether participants’ sexual behavior trajectories are associated with their assigned product utilization trajectory. Finally, COVID-19 might have mitigated participants’ adherence to RM products. Some participants encountered the start of the COVID-19 pandemic during the course of the study. While we minimized interruptions to our trial and ensured that rigor was preserved during the COVID-19 pandemic (Jacobson et al., 2022), we are unable to assess how participants’ sexual behaviors may have changed due to COVID-19 social distancing protocols.

## Conclusion

The current study extends the understanding of three innovative RM modalities by examining self-reported utilization trajectories over time and the characteristics associated with these trajectories. Based on our findings, we encourage others to employ prospective modeling strategies to help characterize different user profiles, pinpoint temporal shifts in usage patterns, and identify psychosocial factors linked to long-term product use. As the RM agenda advances towards an efficacious product, these data will serve to inform future interventions focused on uptake and adherence. These data may allow for just-in-time intervention strategies seeking to support participants’ real-time utilization of and adaptation and adherence to these products. Future studies examining the efficacy of tailored intervention strategies on RM utilization are warranted.

## Data Availability

The data gathered during this study is not publicly available and cannot be shared due to confidentiality, since participants are potentially identifiable from the information contained in the data. Please contact José Bauermeister (bjose@nursing.upenn.edu) to request the data from this study.

## Abbreviations

CASI: Computer-Assisted Self-Interview
DESIRE: Developing and evaluating short-acting innovations for rectal use
HIV: Human immunodeficiency virus
IRB: Institutional Review Board
MSM: Men who have sex with men
MTN: Microbicide trials network
PIN: Personal identification number
PrEP: Pre-exposure prophylaxis
PUEV: Product use end visit
RAI: Receptive anal intercourse
RM: Rectal microbicide
SGM: Sexual and gender minority
SMS: Short messaging service
STI: Sexually transmitted infection
US: United States
